# Resveratrol Modulates Desaturase Expression and Fatty Acid Composition of Cultured Hepatocytes

**DOI:** 10.3389/fnut.2018.00106

**Published:** 2018-11-14

**Authors:** Gianna Kühn, Kathrin Pallauf, Carsten Schulz, Marc Birringer, Beatriz Diaz-Rica, Sonia de Pascual-Teresa, Gerald Rimbach

**Affiliations:** ^1^Institute of Human Nutrition and Food Science, University of Kiel, Kiel, Germany; ^2^Institute of Animal Breeding and Husbandry, University of Kiel, Kiel, Germany; ^3^GMA-Gesellschaft für Marine Aquakultur mbH, Büsum, Germany; ^4^Department of Nutritional, Food, and Consumer Sciences, University of Applied Sciences Fulda, Fulda, Germany; ^5^Department of Metabolism and Nutrition, Institute of Food Science, Food Technology and Nutrition (ICTAN-CSIC), Madrid, Spain

**Keywords:** resveratrol, HepG2 cells, omega-3, desaturase, stearic acid

## Abstract

This study aimed to evaluate whether resveratrol (RSV) and its microbial metabolites dihydro-resveratrol (DHR) and lunularin (LUN) affected fatty acid metabolism and omega-3 polyunsaturated fatty acid (n3-PUFA) synthesis in cultured hepatocytes. To this end, cultured human HepG2 hepatocytes were treated with non-toxic concentrations of these polyphenols (40 μM) and Δ*5-* and Δ*6-desaturase* (*FADS1* and *FADS2*, respectively) expression was measured. Resveratrol induced both genes but DHR and LUN showed no effect. Co-incubation of RSV with α-linolenic acid (ALA) also induced *FADS1* and *FADS2* expression. Moreover, transcription of *carnitine palmitoyltransferase 1A* and *fatty acid synthase* expression was increased, indicating induction of β-oxidation and fatty acid synthesis, respectively. Using gas chromatography to measure fatty acid levels, we observed the impact of RSV with and without ALA treatment on fatty acid composition. However, RSV reduced unsaturated while increasing saturated fatty acid levels. We found lower amounts of monounsaturated fatty acids (16:1n-7c, 18:1n-9c, 18:1n7c, and 20:1n-9) and n3-PUFA docosahexaenoic acid whereas unsaturated fatty acid levels, especially of stearic acid, were elevated. Of interest, once we co-incubated the cells with RSV together with bovine serum albumin, we found no differences in gene expression compared to cells without RSV treatment. Although we found no positive effect of RSV on n3-PUFA synthesis, the stilbene could possibly prevent cellular stress by decreasing unsaturated fatty acid levels.

## Introduction

Consumption of omega-3-fatty-acid-rich foods (1), such as fish, walnuts, or algae (2, 3) may decrease cardiovascular disease risk. The most important omega-3 long-chain polyunsaturated fatty acids (n3-PUFA) are eicosapentaenoic acid (C20:5n-3; EPA) and docosahexaenoic acid (C22:6n-3; DHA). General recommendations of EPA and DHA consumption vary between 250 and 1,000 mg per day for healthy adults (4). Atlantic salmon (*Salmo salar*) contains approximately two grams of long chain PUFAs per 100 g, with farmed fish usually containing higher lipid and thus PUFA levels than wild fish (5). However, aquaculture cannot fully compensate for the decreasing amounts of wild fish and the increasing demand due to growing world population (6). Therefore, developing supplements or functional foods that favor the synthesis of n3-PUFAs from their essential precursor α-linolenic acid (C18:3n-3; ALA) (7) in humans could possibly be an alternative to fish oil consumption *per se*.

**Figure 1:**
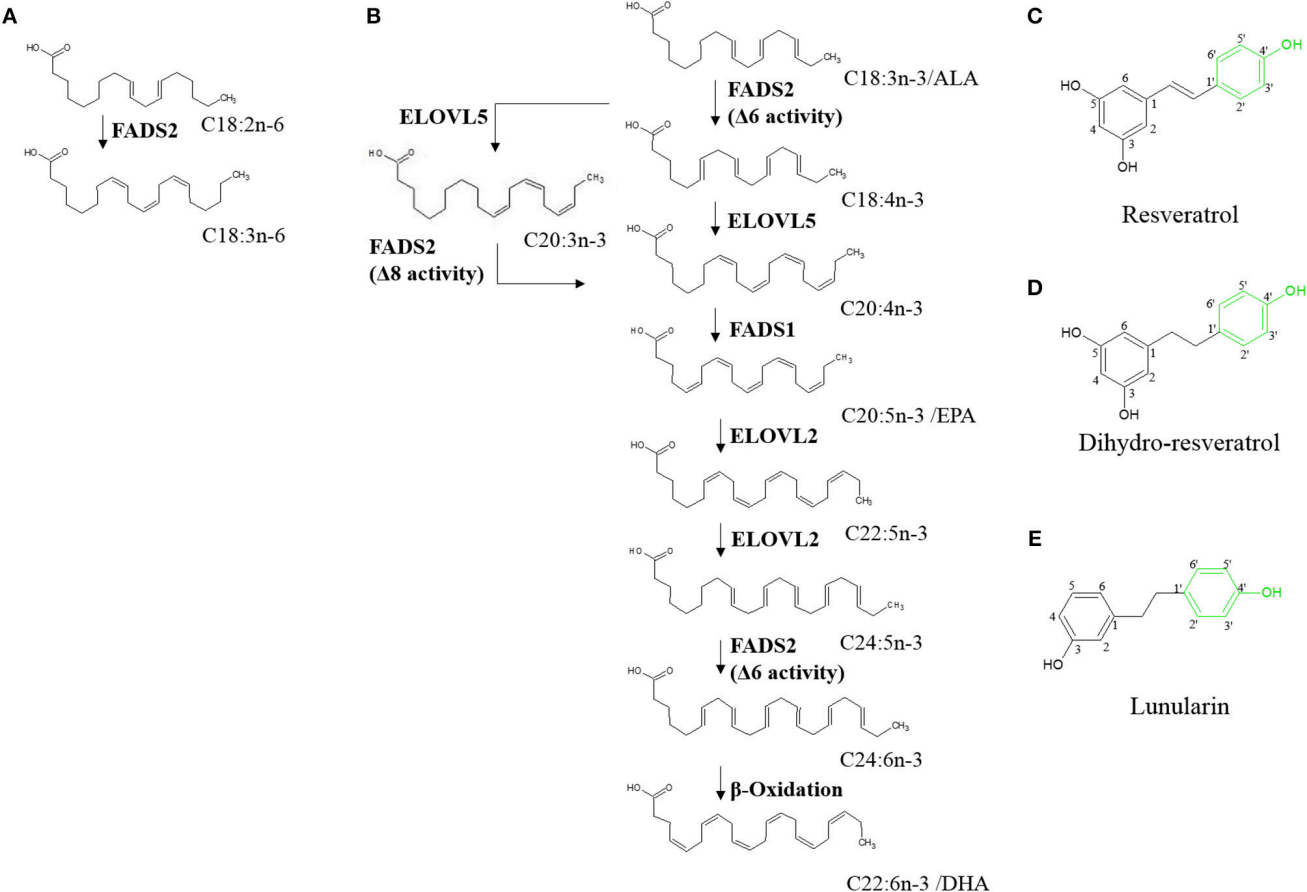
Hepatic endogenous synthesis of long-chain polyunsaturated fatty acids (PUFA) via fatty acid desaturases 1 and 2 (FADS1 and FADS2, respectively) **(A,B)** and test compounds used in the study **(C–E)**. **(A)** step of omega-6 PUFA synthesis regulated via FADS2; **(B)** steps of the two possible pathways of omega-3 PUFA synthesis from α-linolenic acid (C18:3n-3/ALA). α-linolenic acid is either first desaturated by fatty acid desaturase 6 (FADS2) and then elongated by elongase of very long-chain fatty acids 5 (ELOVL5) or first elongated and then desaturated before being desaturated to eicosapentaenoic acid (C20:5n-3/EPA). Synthesis of docosahexaenoic acid (C22:6n-3/DHA) requires further elongation and desaturation and a final peroxisomal step of β-oxidation (8–10). **(C–E)** Chemical structures of resveratrol, dihydro-resveratrol and lunularin. Green marking indicates potentially responsible OH-groups for induction of peroxisome proliferator-activated receptor alpha.

Synthesis of PUFAs (Figure 1) mainly occurs in liver (11). Here, fatty acid desaturases 1 and 2 (FADS1 and FADS2, respectively) insert double bonds into the carbon chains of EPA and DHA precursors. FADS1 adds a double bond to C20:4n-3 whereas FADS2 is responsible for desaturating ALA, C20:3n-3, and C24:5n-3 (12). FADS2 can desaturate at the Δ6- and Δ8-position and thus convert ALA to C18:4n-3 and C20:3n-3 to C20:4n-3 (13, 14). Desaturation at the Δ8-position is exclusively conducted by FADS2 (15).

Transcription factors regulating *FADS1* and *FADS2* gene expression are proliferator-activated receptor alpha (PPARα) (8), liver X receptor and sterol regulatory element binding protein-1c (SREBP-1c) (16). They are regulated by external ligands and cofactors and may induce or inhibit each other (17–20). Moreover, EPA and fasting conditions possibly activate PPARα, whereas C18:1n-9, ALA, C22:5n-3, and DHA impede its action (20). Interestingly and in contrast to regulation of PPARα, EPA and states of hunger have been shown to inhibit SREBP-1c (21). Further PPARα targets have been reported to code for proteins involved in β-oxidation such as carnitine palmitoyltransferase 1A (CPT1) (8). Thereby, the risks because of high levels of unsaturated phospholipids in the cell membrane and, consequently, oxidative damage may be lowered (22, 23).

Resveratrol (RSV) is a plant derived stilbene (Figure 1C) found in grapes and other fruits [reviewed by (24)]. Studies on the polyphenol have reported protective effects against coronary heart diseases [reviewed by (25)], antioxidant activities (26) and other effects that partly mimic those of caloric restriction [reviewed by (27)]. Furthermore, it has been hypothesized that RSV may activate PPARα (via the 4'-OH group; Figure 1C) (28). Thus, RSV may affect n3-PUFA synthesis (29–32).

In the gut, RSV was shown to be metabolized with type and amount of derivatives varying greatly inter- and intraspecifically. In humans, depending on the microbiota composition, RSV is metabolized to dihydro-resveratrol (DHR) and may be further metabolized to lunularin (LUN), both of which contain the potentially PPARα-mediating 4'-OH group (Figures 1D,E) (33). Low concentrations of DHR have been reported as showing proliferative actions whereas higher amounts, similar to RSV, are possibly rather antiproliferative agents (34). Furthermore, low doses of DHR may decrease cholesterol accumulation by inhibiting fatty acid binding protein 4 (35).

In order to compare how RSV and its gut metabolites DHR and LUN affect FADS1 and FADS2 expression, we included all three polyphenols in our experiments. In addition, due to the positive effects reported for RSV on fatty acid metabolism, we aimed to determine the potential of RSV and its derivatives DHR and LUN to modulate fatty acid metabolism and especially n3-PUFA synthesis *in vitro*.

## Materials and methods

### Materials

α-Linolenic acid was obtained from Sigma-Aldrich, Buchs, Switzerland. Resveratrol (IUPAC name: 5-[(E)-2-(4-hydroxyphenyl)ethenyl] benzene-1,3-diol; C_14_H_12_O_3_; CAS number 501-36-0; purity ≥98%) was provided by Carl Roth (Karlsruhe, Germany). Dihydro-resveratrol (IUPAC name: 5-[2-(4-hydroxyphenyl)ethyl] benzene-1,3-diol; C_14_H_14_O_3_; CAS number 58436-28-5; purity ≥98%) was synthesized according to Faragher et al. (36) and LUN (IUPAC name: 3-[2-(4-hydroxyphenyl)ethyl] phenol; C_14_H_14_O_2_; CAS number 37116-80-6; purity >97%) was obtained from Ark Pharm, Inc. (Illinois, USA).

### Cell culture

HepG2 cells (IAZ, Munich, Germany; ATCC No: CCL-23) were cultured in RPMI-1640 with L-glutamine (PAN Biotech, Aidenbach, Germany). The passage number was ≤25. The medium was supplemented with 10% (vol/vol) heat inactivated fetal bovine serum (Gibco™ by Thermo Fisher Scientific GmbH, life technologies™, Darmstadt, Germany) and 1% penicillin/streptomycin (PAN Biotech, Aidenbach, Germany). Cells were grown at 37°C in a humidified 5% CO_2_ incubator in T75 or T175 flasks (37). Cytotoxicity was tested with a neutral red assay (38). To make results comparable, RSV, DHR, and LUN were used at the same non-toxic concentration of 40 μM. Cells were treated at 80% confluence for periods indicated in the subsequent sections. Test compounds were dissolved in dimethyl sulfoxide (DMSO). For co-incubation experiments, 40 mM stock solutions of ALA in EtOH were used to give a final ALA concentration of 50 μM. In all cases, incubations were carried out in duplicate. Experiments were independently performed three times.

### RNA-isolation and quantitative real time (qRT) polymerase chain reaction (PCR)

Cells were treated with 40 μM compounds; in the case of RSV with or without co-incubation with ALA. After 24 h of treatment, cells were harvested with peqGOLD TriFast and RNA was isolated according to the manufacturer's protocol (peqGOLD TriFast, VWR International, Radnor, USA). Sample RNA-concentrations and quality were determined with a Nano Drop 2000 (Thermo Fisher Scientific GmbH, life technologies™, Darmstadt, Germany). The A_260_ was used to calculate RNA concentrations. The ratio of absorbance at 260 and 280 nm was used to assess of RNA purity. Samples were frozen at −80°C at a concentration of 100 ng/μl and the 260/280 ratio was ≥1.8. The ratios for all samples can be found in Supplementary Material. Gene expression was analyzed via quantitative real time polymerase chain reaction (qRT-PCR) with SensiFAST™ SYBR® No-ROX One-Step Kit (Bioline GmbH, Luckenwalde, Germany) using a Rotorgene 6000 cycler (Corbett Life Science, Sydney, Australia). To this end, samples were diluted with nuclease free water and the qRT-PCR reagents to a final concentration of 0.2 ng/μl. Sequences of used primers *FADS1, FADS2, CPT1, fatty acid synthase* (*FASN*), and housekeeping gene *glyceraldehyde-3-phosphate dehydrogenase* (*GAPDH*) are given in Table 1. Primers for *FADS1, FADS2, CPT1*, and *GAPDH* were self-designed using Primer3web. The *FASN* primer was obtained from the Harvard Primer Bank (PrimerBank ID 41872630c1) (39).

**Table 1:** Sequences of primers used in this study.

**Gene name**	**Sequence forward**	**Sequence reverse**
GAPDH—Glyceraldehyde-3-phosphate dehydrogenase	CAATGACCCCTTCATTGACC	GATCTCGCTCCTGGAAGATG
FADS1—fatty acid desaturase 1	GATGCCTCGACACAATTACC	CTGCCCTGACTCCTTTAGTG
FADS2—fatty acid desaturase 2	ACAAGGATCCCGATGTGAAC	TTCGTGCTGGTGATTGTAGG
CPT1—carnitine palmitoyltransferase 1A	CTGCTTTACAGGCGCAAACT	TCATGTGCTGGATGGTGTCT
FASN—fatty acid synthase	AAGGACCTGTCTAGGTTTGATGC	TGGCTTCATAGGTGACTTCCA

The expression levels of the target genes were determined by normalizing the calculated relative concentrations to the housekeeping gene *GAPDH*.

### Western blotting analyses

Cells were treated with 40 μM of RSV with or without ALA for 24 h. For protein analysis, whole cell protein lysates from HepG2 were harvested with RIPA buffer (50 mmol/l Tris, 150 mmol/l NaCl, 0.5% sodium deoxycholate (v/v), 0.1% SDS (w/v), 1% NP-40 (v/v), pH 7.4) supplemented with proteinase inhibitors. Protein concentrations were determined with the Pierce™ BCA assay (Thermo Fisher Scientific GmbH, life technologies™, Darmstadt, Germany) according to the manufacturer's instructions and analyzed by Western Blotting analysis as described before (40). Briefly, 30 μg per samples were heated with loading buffer and applied to Mini-PROTEAN® Stainfree™ Precast Gels (4–20%, BioRad laboratories GmbH, Munich, Germany). Samples were transferred to a polyvinylidenedifluoride membrane (Bio-Rad laboratories GmbH, Munich, Germany) and blocked with skim milk dissolved in Tris-buffered saline + 0.05% (v/v) Tween 20. Membranes were then incubated with the primary antibodies FADS1, FADS2, CPT1, GAPDH (sc-134337, sc-98480, sc-48357, and sc-20357, respectively; Santa Cruz Biotechnology Inc., Dallas, USA) and FASN (bs-5045R, Bioss Inc., Massachusetts, USA). Secondary antibodies were from Santa Cruz Biotechnology Inc., Dallas, USA. Results were analyzed using Image Lab 5.0 (Bio-Rad laboratories GmbH, Munich, Germany).

### Analysis of fatty acid composition

Cells were treated for either 24 or 48 h and harvested by adding 1 mL of Dulbecco's phosphate buffered saline to each well, scratching the cells gently off the plate and transferring the suspension to 2 mL cups, maintaining the samples always on ice. After centrifugation at 2,000 g and 4°C for 5 min, the supernatant was discarded and pellet was resuspended in 200 μl of 0.2M sodium chloride. Samples were frozen at −80°C until further analysis. Fatty acid composition was analyzed according to Vauzour et al. (41) by gas chromatography with a flame ionization detector. Briefly, samples were lyophilized at −60°C for 19 h followed by 1 h at −70°C. Fatty acid methyl esters (FAMEs) were synthesized by incubation with 0.5 M sodium methoxide and incubated at 60°C for 15 min, followed by extraction with acetyl chloride in methanol 1:10 (v/v) and incubation at 60°C for 60 min. One milliliter purified water, 1.5 mL hexan, and sodium sulfate were added to each sample, followed by a centrifugation step (4°C, 1,500 rpm for 5 min). One milliliter of the supernatant was used for further analysis. Samples were evaporated in a Savant™ SPD131DDA SpeedVac™ Concentrator (Thermo Fisher Scientific, Madrid, Spain) and resuspended in hexane. FAMEs analysis was conducted in a 7820A Agilent gas chromatograph (Agilent Technologies Spain) equipped with an Agilent HP-23 capillary column (60 m × 250 μm × 0.25 μm, Agilent Technologies Spain) and helium (1.0 mL/min) as the carrier gas. The temperature protocol was the following: initial temperature 100°C, ramp 8°C/min to 145°C (20 min), ramp 5°C/min to 195°C (5 min), ramp 5°C to 215°C (15 min), ramp 5°C to 230°C (5 min). Chromatograms were recorded and analyzed using Agilent EZChrom Elite software (Agilent Technologies Spain) with 13:0 as the internal standard. Fatty acid composition was calculated as a percentage of the total identified FAMEs. The method was validated with original standards for every fatty acid quantified. Additionally, tridecanoic acid (C13:0) was used as internal standard and in every case a response factor of 1 was used. The limit of quantification was calculated as being below 0.02 mg/g dry matter and the limit of detection was 0.005 mg/mL for every fatty acid.

### Statistics

Statistical analyses were performed with the software RStudio (RStudio, Version 1.0.136, Inc., Boston, USA) (42), using an appropriate mixed model (43, 44). Normal distribution was determined before the analyses with the Shapiro-Wilk-Test and by graphical interpretation using a residual plot. The treatment was regarded as a fixed factor and the replicate as a random factor. Based on this model, a Pseudo *R*^2^ was calculated (45) and an analysis of variances (ANOVA) was conducted, followed by a *post-hoc* multiple comparison test of Dunnett (46) to compare the the effects to the DMSO control or a *post-hoc* multiple comparison test of Tukey to determine differences between the means of all treatments.

## Results

Compound cytotoxicity was tested in a neutral red assay (Supplementary Figure S1) and only a non-toxic dose was applied in the following experiments.

**Figure 2:**
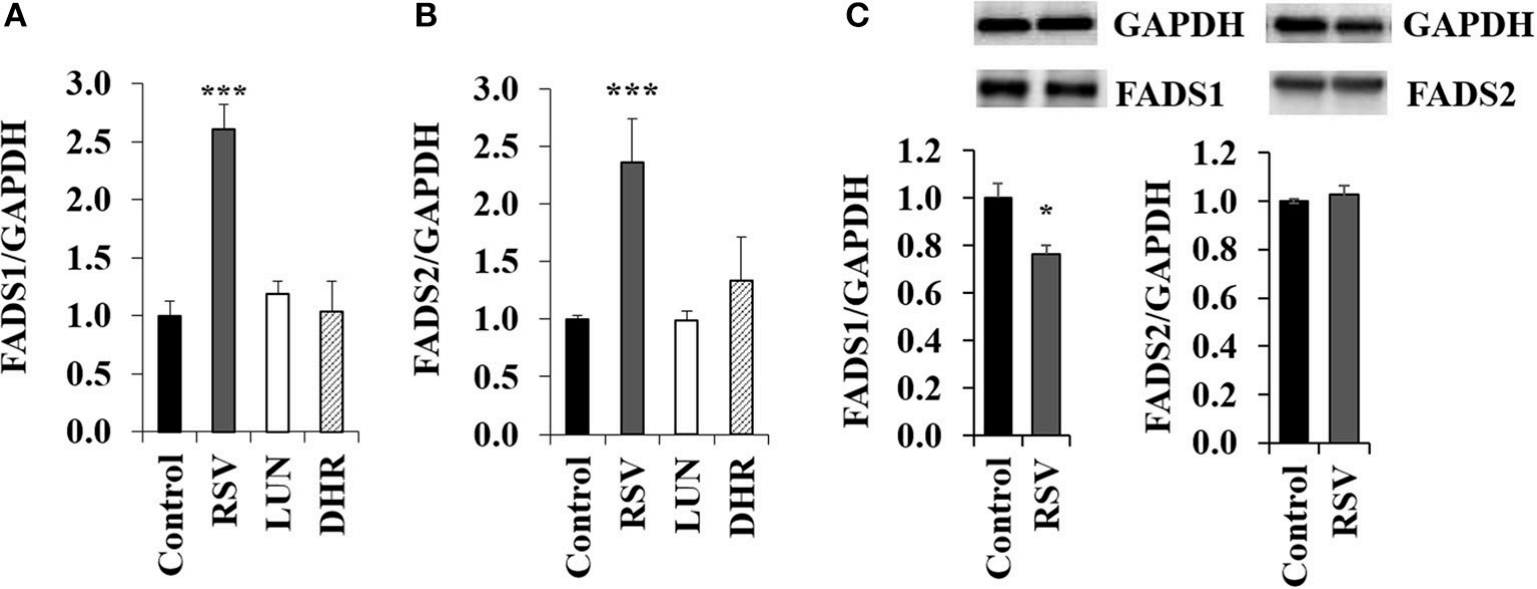
Effects of treating HepG2 for 24 h with 40 μM of resveratrol (RSV), lunularin (LUN) or dihydro-resveratrol (DHR) on the mRNA level of **(A)** fatty acid desaturase 1 (FADS1) and **(B)** fatty acid desaturase 2 (FADS2). Values are relative to the solvent control DMSO set to be 1.0. Expression levels were normalized to glyceraldehyde 3-phosphate dehydrogenase (GAPDH) and are shown as means + standard error (*n* = 3; ^*^ *P* ≤ 0.05, ^***^P ≤ 0.001; with *post-hoc* multiple comparison test of Dunnett). Effects of treating HepG2 for 24 h with 40 μM of resveratrol on protein levels of FADS1 and FADS2 **(C)**, analyzed by Western Blotting analysis. Protein levels were normalized to the housekeeping protein GAPDH.

Treating HepG2 cells with 40 μM of RSV induced transcription of *FADS1* and *FADS2* while 40 μM of DHR and LUN showed no effect (Figures 2A,B). On the protein level, RSV slightly lowered FADS1 and did not have any impact on FADS2 (Figure 2C; Supplementary Figure S3).

**Figure 3:**
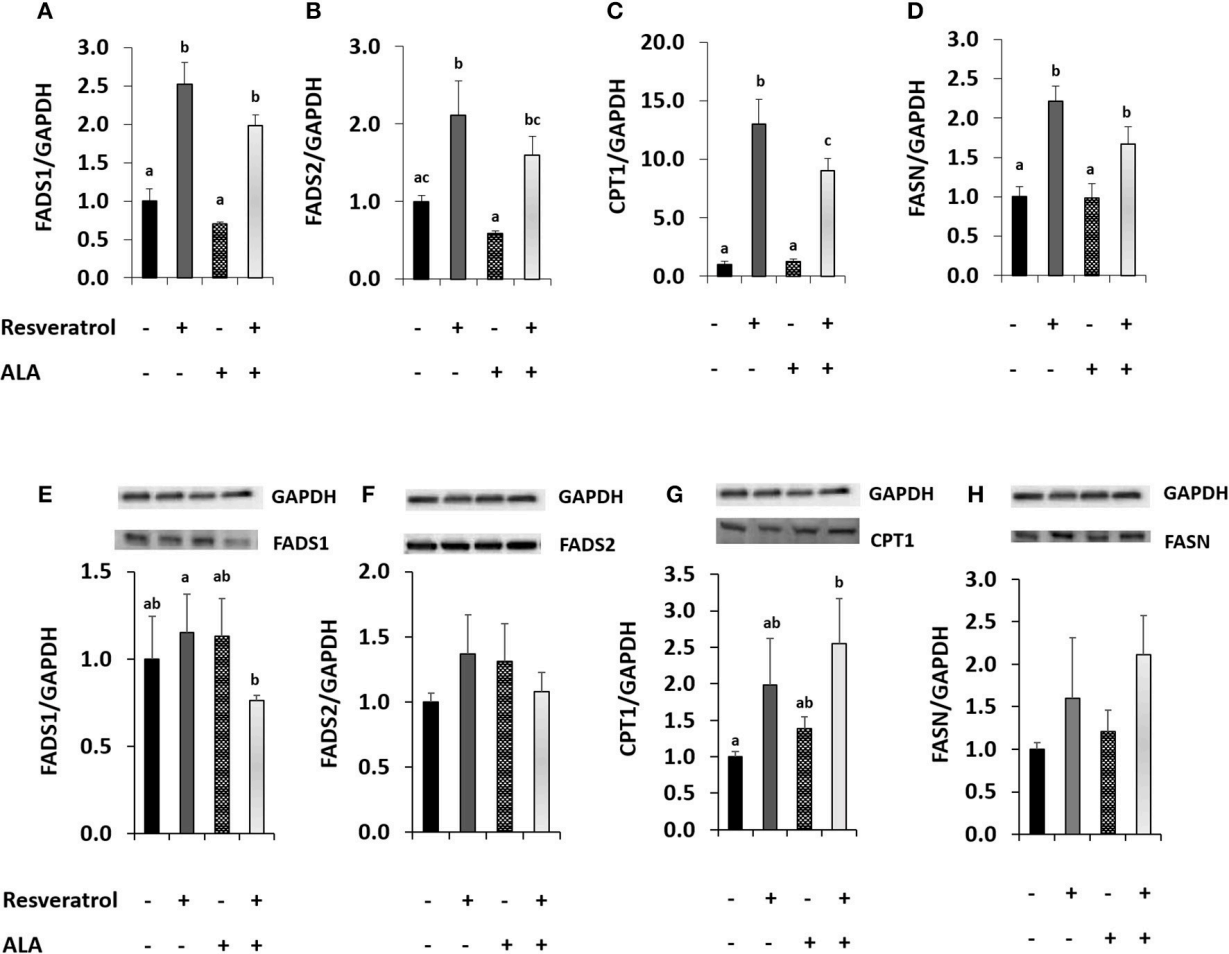
Effects of treating HepG2 for 24 h with 40 μM of resveratrol and 50 μM α-linolenic acid (ALA) in ethanol (EtOH) or 0.125% EtOH + 0.04% DMSO solvent control on **(A)** fatty acid desaturase 1 (FADS1), **(B)** fatty acid desaturase 2 (FADS2), **(C)** carnitine palmitoyltransferase 1A (CPT1), and **(D)** fatty acid synthase (FASN) mRNA levels. Values are relative to DMSO + EtOH control, which was set to be 1.0. Expression levels were normalized to the housekeeping gene glyceraldehyde 3-phosphate dehydrogenase (GAPDH). *n* = 3; mean + standard error. Values with different superscript letters differ significantly with *p*-values < 0.05; with *post-hoc* multiple comparison test of Tukey. Effects of treating HepG2 for 24 h with 40 μM of resveratrol and 50 μM ALA in EtOH or EtOH + DMSO solvent control on protein levels of FADS1, FADS2, CPT1, or FASN **(E-H)**, analyzed by Western Blotting analysis. Protein levels were normalized to the housekeeping protein GAPDH.

We conducted a second experiment to determine whether application of external ALA influenced RSV action. In our first attempt, we applied ALA complexed with BSA. When applying RSV together with BSA, the induction of *FADS1* and *FADS2* disappeared (Supplementary Figures S2, S4), possibly because of RSV binding to BSA (47). Therefore, we chose to apply ALA dissolved in EtOH for our experiments of co-incubation with RSV. When adding EtOH-dissolved ALA, *FADS2* expression was inhibited compared to the EtOH solvent control. Induction of *FADS1* and *FADS2* after 40 μM RSV co-incubation with 50 μM of ALA (Figures 3A,B) was almost as strong as when applying RSV alone (Figures 2A,B). However, protein analysis showed a tendency toward lower FADS1 levels due to treatment with RSV and ALA (Figures 3E,F; Supplementary Figure S5).

Resveratrol-induced *CPT1* transcription was more than 10-fold higher than in the solvent control and remained around 9-fold higher when comparing ALA with ALA and RSV (Figure 3C). Resveratrol also induced *FASN* (Figure 3D). Levels of both proteins appeared to be elevated by RSV and ALA treatment in Western blots, albeit effects on FASN were not significant (Figures 3G,H).

Determining fatty acid composition with gas chromatography, we found that in cells treated with RSV and ALA, stearic acid and total amounts of saturated fatty acids were enhanced. In contrast, levels of monounsaturated fatty acids C16:1n-7, C18:1n-9c, and C18:1n-7c were decreased. Total levels of PUFA were affected due to the addition of ALA but not the treatment with RSV. While α-linolenic acid levels tended to and amounts of C20:3n-3 were increased, C22:5n-3 and DHA levels were reduced compared to non-ALA treated cells. With exception of control ALA treated cells, EPA levels were under the detectable limit. Fatty acid composition after 24 and 48 h was very similar (Tables 2, 3).

**Table 2:** Fatty acid composition (in % of total fatty acid methyl esters) of HepG2 cells treated for 24 h with 40 μM of resveratrol (RSV) and 50 μM of α-linolenic acid (ALA) dissolved in ethanol (EtOH) or solvent control EtOH + DMSO.

**Fatty acid**	**Control**	**RSV 40 μM**	**Control + ALA**	**RSV 40 μM + ALA**
14:0	2.08 ± 0.28	2.07 ± 0.22	2.09 ± 0.11	1.84 ± 0.20
16:0	21.28 ± 0.33	23.28 ± 0.51	21.99 ± 0.79	24.96 ± 4.66
**18:0**	**7.74 ±0.24^a^**	**15.11 ±0.90^b^**	**7.95 ±0.09^a^**	**13.87 ±0.88^c^**
20:0	0.35 ± 0.06	0.55 ± 0.14	0.32 ± 0.08	1.08 ± 0.97
**Sum SFA**	**31.44 ±0.14^a^**	**41.02 ±1.20^b^**	**32.36 ±0.73^a^**	**41.75 ±4.99^b^**
16:1n-7	8.61 ± 0.41^a^	5.37 ± 0.24^b^	7.59 ± 0.60^c^	4.08 ± 0.67^d^
18:1n-9c	28.13 ± 0.93^a^	24.86 ± 0.24^b^	24.42 ± 2.08^b^	20.38 ± 2.77^c^
18:1n-7c	17.70 ± 1.13^a^	13.45 ± 0.61^b^	13.96 ± 1.71^b^	11.00 ± 2.47^c^
20:1n-9	1.86 ± 0.17^a^	1.75 ± 0.05^a^	1.47 ± 0.13^b^	1.42 ± 0.23^b^
**Sum MUFA**	**56.30 ±2.48^a^**	**45.43 ±0.53^b^**	**47.44 ±4.50^b^**	**36.88 ±6.09^c^**
18:2n-6c	1.33 ± 0.16^ac^	1.41 ± 0.11^ac^	1.21 ± 0.08^b^	1.74 ± 0.17^c^
18:3n-3	nd	nd	3.03 ± 1.88^a^	6.76 ± 1.34^b^
20:4n-6	3.67 ± 0.25^ab^	3.81 ± 0.26^a^	3.27 ± 0.21^ab^	3.11 ± 0.47^b^
**20:3n-3**	**2.61 ±2.01^a^**	**3.30 ±1.02^a^**	**4.19 ±2.96^ab^**	**5.85 ±1.85^b^**
20:4n-3	nd	nd	0.77 ± 0.12	nd
**20:5n-3**	**nd**	**nd**	**2.43 ±0.19**	**nd**
22:5n-3	0.59 ± 0.08^ad^	0.77 ± 0.16^bd^	1.02 ± 0.07^c^	0.69 ± 0.10^d^
**22:6n-3**	**3.28 ±0.02^a^**	**3.28 ±0.05^a^**	**3.59 ±0.16^a^**	**2.68 ±0.53^b^**
Sum PUFA	9.65 ± 2.43^a^	10.13 ± 1.42^a^	16.00 ± 5.15^b^	15.34 ± 1.63^b^
EPA/DHA	nv	nv	0.68 ± 0.08	nv
ALA/LA	nv	nv	2.51 ± 1.40^a^	4.99 ± 1.05^b^

*n = 3; mean ± standard deviation. Values with different superscript letters within one row significantly differ with p-values < 0.05 with post-hoc multiple comparison test of Tukey. SFA: saturated fatty acids; MUFA: monounsaturated fatty acids; PUFA: polyunsaturated fatty acids; nd: not detectable (values below detection limits); nv: no value (due to values below detection limits). Bold values indicate most important significant findings*.

**Table 3:** Fatty acid composition (in % of total fatty acid methyl esters) of HepG2 cells treated for 48 h with 40 μM of resveratrol (RSV) and 50 μM of α-linolenic acid (ALA) dissolved in ethanol (EtOH) or solvent control EtOH + DMSO.

**Fatty acid**	**Control**	**RSV 40 μM**	**Control + ALA**	**RSV 40 μM + ALA**
14:0	1.97 ± 0.31	1.96 ± 0.20	2.23 ± 0.12	2.09 ± 0.19
16:0	21.27 ± 1.07^a^	23.68 ± 0.84^b^	22.95 ± 0.12^abc^	23.22 ± 1.64^c^
**18:0**	**7.53 ±0.39^a^**	**17.07 ±1.43^b^**	**8.36 ±0.14^a^**	**15.73 ±1.88^b^**
20:0	0.34 ± 0.13^a^	0.58 ± 0.12^b^	0.27 ± 0.14^a^	0.62 ± 0.02^b^
**Sum SFA**	**31.11 ±1.59^a^**	**43.29 ±2.27^b^**	**33.82 ±0.29^a^**	**41.66 ±3.49^b^**
16:1n-7	8.33 ± 0.47^a^	4.53 ± 0.24^b^	7.77 ± 0.48^a^	3.65 ± 0.29^c^
18:1n-9c	28.46 ± 1.26^a^	25.09 ± 0.42^b^	26.05 ± 1.39^c^	21.55 ± 0.84^d^
18:1n-7c	19.96 ± 2.23^a^	12.75 ± 0.60^b^	15.87 ± 1.59^b^	11.04 ± 0.74^c^
20:1n-9	1.79 ± 0.05^a^	1.61 ± 0.09^a^	1.63 ± 0.08^a^	1.34 ± 0.19^b^
**Sum MUFA**	**58.54 ±3.98^a^**	**43.99 ±0.31^b^**	**51.32 ±3.49^c^**	**37.59 ±1.37^d^**
18:2n-6c	1.15 ± 0.56^a^	1.74 ± 0.68^a^	0.93 ± 0.12^a^	2.39 ± 1.42^b^
18:3n-3	*nd*	*nd*	0.98 ± 0.52^a^	5.36 ± 2.05^b^
20:4n-6	2.77 ± 0.18^a^	3.25 ± 0.23^b^	2.59 ± 0.26^b^	2.88 ± 0.24^c^
**20:3n-3**	**4.61 ±3.40^a^**	**3.60 ±2.15^a^**	**3.04 ±1.44^a^**	**6.39 ±2.53^b^**
20:4n-3	nd	nd	0.50 ± 0.18	nd
**20:5n-3**	**nd**	**nd**	**2.11 ±0.53**	**nd**
22:5n-3	0.29 ± 0.06^ad^	0.61 ± 0.15^b^	0.88 ± 0.16^c^	0.39 ± 0.29^d^
**22:6n-3**	**2.59 ±0.20^a^**	**2.79 ±0.27^a^**	**3.37 ±0.38^b^**	**2.53 ±0.22^a^**
Sum PUFA	7.28 ± 4.51^a^	9.12 ± 2.23^ab^	11.83 ± 3.23^b^	14.28 ± 4.76^c^
EPA/DHA	nv	nv	0.63 ± 0.12	nv
ALA/LA	nv	nv	1.06 ± 0.44^a^	2.24 ± 1.84^b^

*n = 3; mean ± standard deviation. Values with different superscript letters within one row significantly differ with p-values < 0.05 with post-hoc multiple comparison test of Tukey. SFA: saturated fatty acids; MUFA: monounsaturated fatty acids; PUFA: polyunsaturated fatty acids; nd: not detectable (values below detection limits); nv: no value (due to values below detection limits). Bold values indicate most important significant findings*.

When complexing ALA with BSA (Supplementary Tables S1, S2), the impact of RSV was similar albeit less strong than with EtOH dissolved ALA. In both experiments, total lipid contents did not differ significantly between the treatments (data not shown).

## Discussion

Resveratrol but not its metabolites LUN or DHR induced the expression of *FADS1* and *FADS2* when used at a concentration of 40 μM. While the 4′-OH group in RSV was shown to be important for PPARα activation (28), cell cycle and antioxidant effects potentially further depend on the 3- and 5-OH groups and the important *trans* double bond in the stilbenic skeleton (48). Thus, the missing impacts of LUN and DHR on *FADS1* and *FADS2* transcription may be due to the saturated instead of trans-unsaturated bond in both compounds and, additionally, the absence of a 3-OH group in the structure of LUN (Figures 1C–E). Furthermore, it has to be considered that we did not measure intracellular concentrations of the test compounds and therefore cannot exclude lowered bioavailability of LUN and DHR compared to RSV.

ALA-addition to the cells did not significantly change FADS1 or FADS2 protein levels compared to non-ALA treated cells with or without RSV.

However, as can be seen in the fatty acid analyses (Tables 2, 3), ALA appears to be taken up by the cells. This coincides with a previously reported dose-dependent increase of intracellular ALA after ALA co-treatment (49). Furthermore, RSV treatment increased ALA levels even more, which has been found in rat hepatocytes treated with 50 μM RSV (30) and Atlantic salmon supplemented with 2.5 g/kg RSV (32).

Interestingly, in the cells, levels of total saturated fatty acids, especially stearic acid, were increased by RSV treatment, whereas amounts of monounsaturated fatty acids were lowered. Application of RSV and ALA increased C20:3n-3 levels while lowering EPA, C22:5n-3, and DHA levels. Eicosatetraenoic acid (C20:4n-3) was not detectable in cells treated with RSV and ALA. Based on our mRNA data for *FADS2*, we did not expect a decrease of fatty acids that are synthesized by this gene product. However, enhanced levels of C20:3n-3 but non-detectable levels of C20:4n-3 after application with RSV and ALA may be caused by decreased FADS2/Δ8-desaturase activity (1) or increased β-oxidation (2):

1. The initial elongation of ALA may be initiated but activity of FADS2 may be inhibited by RSV. As a multi-pass membrane protein, FADS2 (50) possibly interacts with RSV which would be expected to accumulate in membrane departments due to its low solubility in aqueous environments. If RSV somehow inhibited FADS2, it might not be able to act at the Δ6-position and instead, ELOV5 might induce the alternative pathway of synthesis of n3-PUFA (13). However, Δ8-desaturase activity of FADS2 may also be repressed, making C20:3n-3 potentially an inhibitor of further fatty acid desaturation and elongation (Figure 1). Additionally, it has been hypothesized that animals lack Δ8-desaturase activity (51). Missing C20:4n-3 and no effects on FADS2 protein levels after treatment with RSV and EtOH-dissolved ALA supports the theory of missing or blocked Δ8-desaturase activity. This may explain, why C20:3n-3 accumulates in cells treated with ALA, although mRNA level of *FADS2* is elevated.
  In contrast, detectable levels of C20:4n-3 being the product of C20:3n-3 desaturation and C18:4n-3 elongation in control samples indicate an induction and execution of the pathway of n3-PUFA synthesis in the absence of RSV. In accordance with this hypothesis of inhibited n3-PUFA synthesis after C18:3n-3 elongation in RSV-treated cells, EPA, C22:5n-3, and DHA levels were lowered in these samples.
2. Another explanation may be an increased β-oxidation and thereby degradation of mono- and polyunsaturated fatty acids. Potential induction of β-oxidation is indicated by enhanced *CPT1* expression and protein levels, CPT1 being essential for the passage of fatty acids through the mitochondrial membrane (52). As reported before (53), we found RSV to highly increase *CPT1* expression. Thus, via β-oxidation, unsaturated fatty acids may directly be metabolized to saturated fatty acids (54). Additionally, unsaturated fatty acids may be broken down to acetyl CoA, which in turn can be used as a precursor for *de novo* synthesis of saturated fatty acids (55). *De novo* synthesis of fatty acids seems to be induced by RSV, since we found it to increase the expression and protein levels of FASN, which is central in fatty acid synthesis (56).

In contrast to PUFAs, saturated fatty acids are rather related to increased inflammation and high low density lipoprotein cholesterol levels (57, 58). However, it has been hypothesized that stearic acid may improve fitness and lower free fatty acid levels in fruit flies (59). In laboratory mice, dietary stearic acid was reported as reducing visceral adipose tissue. The authors related the effect to apoptosis induction of fat cells due to stearic acid application, which they confirmed *in vitro* in cultured preadipocytes (60). Furthermore, stearic acid may also decrease cholesterol absorption and inflammatory response (61, 62).

Resveratrol may reduce inflammation by lowering the levels of high-sensitivity C-reactive protein and CC-chemokine ligand 5 *in vivo* (63, 64). In human plasma, these markers were found to be modulated by different fatty acids. Stearic acid and C18:2n-6, which we found to be increased by RSV treatment (Tables 2, 3), may also lower high-sensitivity C-reactive protein and CC-chemokine ligand 5 levels [reviewed by Hunter et al. (65)]. Resveratrol-induced C18:2n-6 increase is in line with previous results in Atlantic salmon (32). The positive effects reported for RSV on cholesterol levels and inflammation (66, 67) may hypothetically, in part, be related to its alteration of fatty acid profiles. Additionally, C16:1n-7, C18:1n-9c, and C18-1n-7c, which were decreased by RSV (Tables 2, 3), have been related positively to the induction of inflammatory enzymes, such as high-sensitivity C-reactive protein and CC-chemokine ligand 5 (62).

An increase of C20:3n-3 may lower inflammation since it has been reported as dose dependently inhibiting inflammatory markers. Of interest, this n3-fatty acid was shown to improve skin barrier function and health in UV-irradiated hairless mice, possibly via the nuclear factor kappa-light-chain-enhancer of activated B cells pathway (68). However, C20:3n-3 has a lower anti-inflammatory potential than EPA (69).

Since unsaturated carbon chains of n3-PUFA enhance susceptibility to oxidative damage (23), RSV may hypothetically protect cells by lowering levels of EPA, C22:5n-3, and DHA. Of interest, RSV has been shown to induce effects similar to caloric restriction [reviewed by Pallauf et al. (27)]. One of the putatively life-span prolonging mechanisms, which RSV and caloric restriction share, could possibly be lowering unsaturated FA content of membrane lipids (70). If such a mechanism contributed to lifespan extension, one could possibly explain species-dependent effects of RSV on longevity, since saturation indexes of membrane very between species [reviewed by Hector et al. (24)].

The increase of *FADS1* and *FADS2* expression may be due to RSV inducing their transcription via PPARα (8, 71). Conversely, although we found elevated mRNA levels of *FADS1* and *FADS2* after RSV treatment, neither protein levels nor fatty acid concentrations of n3-PUFA were elevated after 24 or 48 h. We cannot exclude a long-term enhancing effect of RSV on n3-PUFA levels. However, decreased EPA due to RSV treatment but no influence on *FADS* expression was reported before for salmon fed 1.5 g RSV per kg feed (32). This was in contrast to previous findings in trout fed 0.3% dry matter RSV (increased n3-PUFA but no effect on *FADS2*) and rat hepatocytes exposed to 50 μM RSV (increased n3-PUFA in phospholipids) (30, 31), indicating potential species- and/or dose-specific effects.

It needs to be considered that concentrations of the test compounds, as used in the present cell culture study, were manifold higher than plasma concentrations reported in humans (72).

In conclusion, albeit application of 40 μM RSV to human hepatocytes increased the expression of genes involved in n3-PUFA synthesis, levels of EPA and DHA were reduced. Resveratrol, furthermore, led to elevated amounts of stearic acid, a finding, which should be validated in studies in laboratory rodents and humans. RSV metabolites LUN and DHR did not show an impact on FADS1 or FADS2.

## Data availability

The raw data of this study may be requested of the corresponding author of this article.

## Author contributions

The experimental work was conducted by GK and BD-R under the supervision of KP, GR, and SdP-T. MB synthesized the test compound dihydro-resveratrol. The study was designed by GR, KP and CS. All authors contributed to the methods and interpretation of the results. The text was written by GK, KP, and GR and revised by the other authors. All authors have given approval to its final version.

### Conflict of interest statement

CS was employed by Gesellschaft für Marine Aquakultur mbH (Büsum, Germany). The remaining authors declare that the research was conducted in the absence of any commercial or financial relationships that could be construed as a potential conflict of interest.

## Abbreviations

ALA: α-linolenic acid
BSA: bovine serum albumin
CPT1: carnitine palmitoyltransferase 1A
DHA: docosahexaenoic acid
DHR: dihydro-resveratrol
EPA: eicosapentaenoic acid
EtOH: ethanol
FADS: fatty acid desaturase
FAME: Fatty acid methyl ester
FASN: fatty acid synthase
GAPDH: glyceraldehyde 3-phosphate dehydrogenase
n3-PUFA: omega-3 polyunsaturated fatty acid
LUN: lunularin
PPAR: peroxisome proliferator-activated receptor
RSV: resveratrol
SREBP-1c: sterol regulatory element binding protein-1c.
