# Supplementary material for: Resveratrol Modulates Desaturase Expression and Fatty Acid Composition of Cultured Hepatocytes
Source: Front Nutr. 2018 Nov 14;5:106. doi: 10.3389/fnut.2018.00106 (PMC6246710; doi:10.3389/fnut.2018.00106)
Supplement: Supplementary file 1 [file Data_Sheet_1.PDF]

Supplementary Material:

Resveratrol modulates desaturase expression and fatty acid composition of cultured hepatocytes.

1 SUPPLEMENTARY METHODS

260/280 absorbance ratios of RNA samples used for PCR

Samples used in Fig. 2

Control I – 1.91
Control II – 1.82
Control III – 1.88
Control IV – 1.86
Control V – 1.85
Control VI – 1.83
Resveratrol I – 1.83
Resveratrol II – 1.83
Resveratrol III – 1.84
Resveratrol IV – 1.86
Resveratrol V – 1.83
Resveratrol VI – 1.84
Lunularin I – 1.89
Lunularin II – 1.89
Lunularin III – 1.86
Lunularin IV – 1.85
Lunularin V – 1.85
Lunularin VI – 1.83
Dihydro-Resveratrol I – 1.87
Dihydro-Resveratrol II – 1.89
Dihydro-Resveratrol III – 1.91
Dihydro-Resveratrol IV – 1.90
Dihydro-Resveratrol V – 1.81
Dihydro-Resveratrol VI – 1.81

Samples used in Fig. 3

Control Ethanol I – 1.82
Control Ethanol II – 1.81
Control Ethanol III – 1.81
Control Ethanol IV – 1.83
Control Ethanol V – 1.84
Control Ethanol VI – 1.82
Resveratrol Ethanol I – 1.80
Resveratrol Ethanol II – 1.83
Resveratrol Ethanol III – 1.84
Resveratrol Ethanol IV – 1.81

Resveratrol Ethanol V – 1.82
Resveratrol Ethanol VI – 1.81
Control ALA I – 1.80
Control ALA II – 1.82
Control ALA III – 1.85
Control ALA IV – 1.88
Control ALA V – 1.82
Control ALA VI – 1.83
Resveratrol ALA I – 1.81
Resveratrol ALA II – 1.80
Resveratrol ALA III – 1.84
Resveratrol ALA IV – 1.82
Resveratrol ALA V – 1.87
Resveratrol ALA VI – 1.81

Samples used in Fig. 4
Control BSA I – 1.87
Control BSA II – 1.86
Control BSA III – 1.82
Control BSA IV – 1.81
Control BSA V – 1.80
Control BSA VI – 1.86
Resveratrol BSA I – 1.85
Resveratrol BSA II – 1.85
Resveratrol BSA III – 1.81
Resveratrol BSA IV – 1.81
Resveratrol BSA V – 1.80
Resveratrol BSA VI – 1.86
Control ALA I – 1.89
Control ALA II – 1.88
Control ALA III – 1.88
Control ALA IV – 1.86
Control ALA V – 1.81
Control ALA VI – 1.83
Resveratrol ALA I – 1.81
Resveratrol ALA II – 1.83
Resveratrol ALA III – 1.85
Resveratrol ALA IV – 1.83
Resveratrol ALA V – 1.83
Resveratrol ALA VI – 1.81

Supplementary cell culture
For the study of RSV and BSA-complexed ALA co-treatment, medium was supplemented with 40 µM of RSV or DMSO (RSV-solvent control) and, additionally, 50 µM BSA-bound ALA or 13 mg/ml BSA (equaling the concentration of BSA in 50µM BSA-bound ALA).

To this end, α-linolenic acid was complexed with bovine serum albumin (BSA) by diluting it 160mM in Ethanol (EtOH), evaporating the EtOH under a nitrogen flux, and diluting it in potassium hydroxide solution. After heating at 70°C for 1h, BSA dissolved in Dulbecco's phosphate buffered saline (pH 7.2) was added. Samples were incubated at 37°C for 48h and diluted in Dulbecco's phosphate buffered saline to obtain a final concentration of ALA-stock of 360 µM (Wiesenfeld et al., 2001). BSA/Dulbecco's phosphate buffered saline control solution (13mg/ml) without ALA was prepared similarly in parallel.

2 SUPPLEMENTARY RESULTS

2.1 SUPPLEMENTARY FIGURES

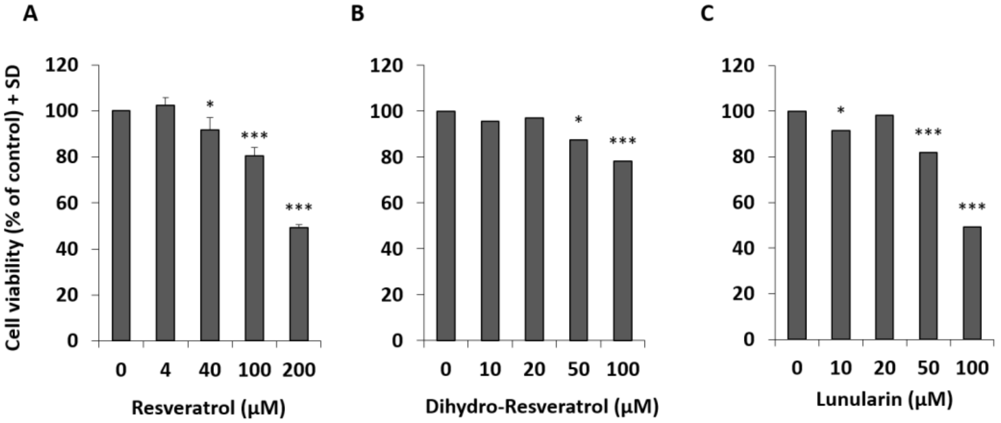

FIGURE S1 | Results of neutral red assay. HepG2 cells were treated with indicated concentrations and substances for 24 hours. Substances were dissolved in DMSO, which was as control. Cell viability is calculated as the percentage of the DMSO control (%). Results are mean values + SD of n=2 in triplicate.

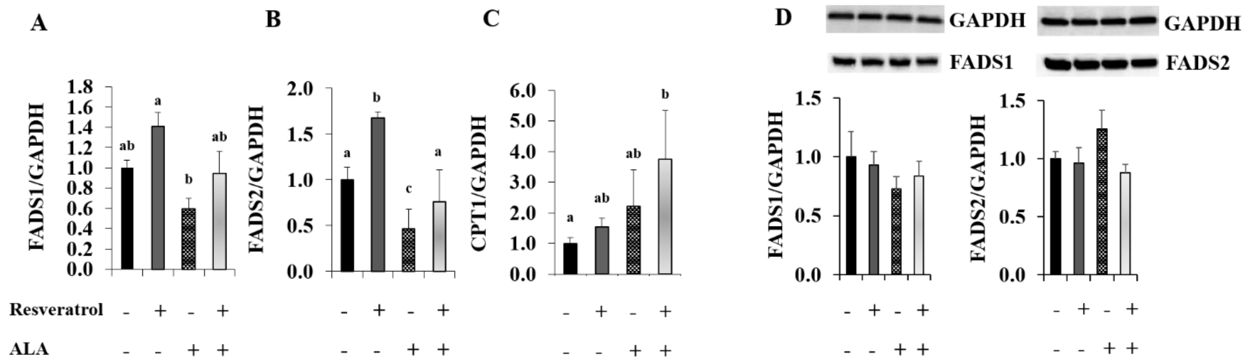

FIGURE S2 | Effects of treating HepG2 for 24h with 40 µM of resveratrol and additional 50 µM of BSA-bound α-linolenic acid (ALA) or 13 mg/ml of BSA on the mRNA level of **(A)** fatty acid desaturase 1 (FADS1), **(B)** fatty acid desaturase 2 (FADS2) and **(C)** carnitine palmitoyltransferase 1A (CPT1). Values of resveratrol are relative to the solvent control set to be 1.0. Expression levels were normalized to the housekeeping gene glyceraldehyde 3-phosphate dehydrogenase (GAPDH). n=3; mean + standard error. Values with different superscript letters within one row significantly differ with p-values < 0.05; with post-hoc multiple comparison test of Tukey. Effects of treating HepG2 for 24h with 40µM of resveratrol and additional 50 µM of BSA-bound ALA or 13 mg/ml of control BSA on protein levels of FADS1 and FADS2 **(D)**, analyzed by Western Blotting analysis. Protein levels were normalized to the housekeeping protein glyceraldehyde 3-phosphate dehydrogenase (GAPDH).

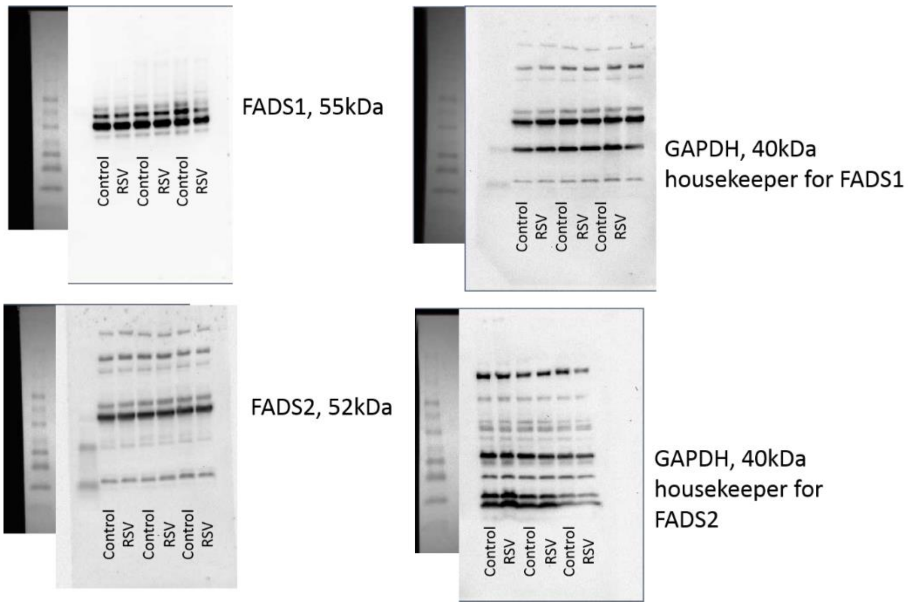

FIGURE S3 | Effects of treating HepG2 for 24h with 40µM of resveratrol (RSV) on protein levels of fatty acid desaturase 1 (FADS1) and FADS2, analyzed by Western Blotting analysis. Protein levels were normalized to the housekeeping protein glyceraldehyde 3-phosphate dehydrogenase (GAPDH). Blots show three independent repetitions n=3.

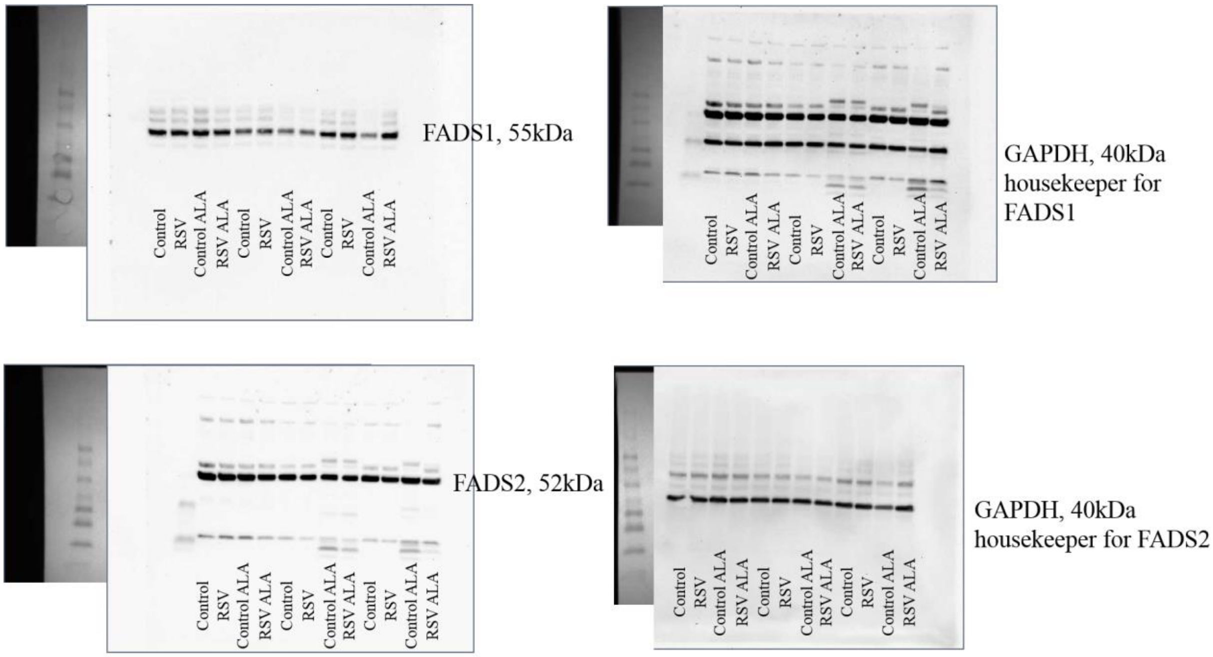

Figure S4 | Effects of treating HepG2 for 24h with 40µM of resveratrol and additional 50 µM of BSA-bound α-linolenic acid (ALA) or 13 mg/ml of control BSA on protein levels of fatty acid desaturase 1 (FADS1) and FADS2, analyzed by Western Blotting analysis. Protein levels were normalized to the housekeeping protein glyceraldehyde 3-phosphate dehydrogenase (GAPDH). Blots show three independent repetitions n=3.

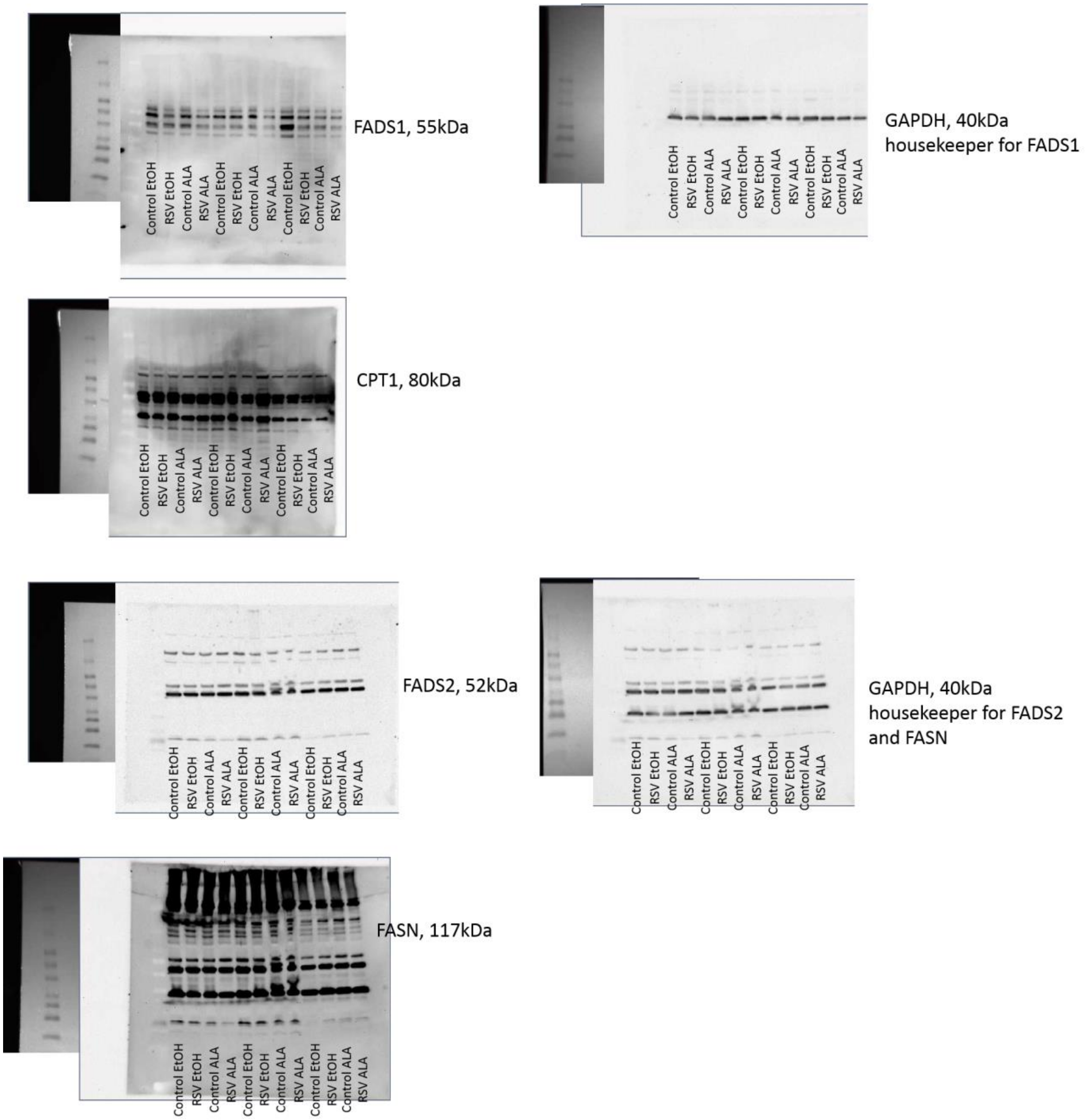

Figure S5 | Effects of treating HepG2 for 24h with 40µM of resveratrol (RSV) and 50 µM α-linolenic acid (ALA) in ethanol (EtOH) or 0.125% EtOH, 0.04% DMSO solvent control on protein levels of fatty acid desaturase 1 (FADS1), carnitine palmitoyl transferase 1 (CPT1), FADS2, and fatty acid synthase (FASN) analyzed by Western Blotting analysis. Protein levels were normalized to the housekeeping protein glyceraldehyde 3-phosphate dehydrogenase GAPDH. Blots show three independent repetitions n=3.

2.2 SUPPLEMENTARY TABLES

Table S1 | Fatty acid composition (in % of total fatty acid methyl esters) of HepG2 cells treated for 24h with 40 µM of resveratrol (RSV) and additional 50 µM of BSA-bound *α*-linolenic acid (ALA) or 13 mg/ml of control BSA.

Fatty acid	Control	RSV 40µM	Control + ALA	RSV 40µM + ALA
14:0	1.68 ± 0.18^{a}	1.43 ± 0.05^{ab}	1.35 ± 0.65^{ab}	0.98 ± 0.26^{b}
16:0	20.43 ± 0.81^{a}	20.41 ± 1.45^{a}	15.73 ± 2.55^{b}	14.47 ± 0.47^{b}
18:0	5.82 ± 0.50^{a}	12.19 ± 1.12^{b}	7.81 ± 0.08^{c}	8.40 ± 0.70^{ac}
20:0	0.71 ± 0.11^{a}	0.67 ± 0.19^{a}	0.30 ± 0.04^{bc}	0.37 ± 0.12^{c}
Sum SFA	32.35 ± 13.10^{a}	34.70 ± 11.00^{a}	25.19 ± 5.89^{b}	24.21 ± 4.41^{b}
16:1n-7	5.82 ± 0.80^{a}	4.58 ± 0.62^{b}	3.32 ± 0.80^{c}	2.36 ± 0.05^{d}
18:1n-9c	27.15 ± 1.39^{a}	27.65 ± 0.93^{a}	15.01 ± 1.05^{b}	14.51 ± 0.22^{bc}
18:1n-7c	15.83 ± 1.68^{a}	14.25 ± 1.75^{a}	8.28 ± 1.58^{b}	7.93 ± 0.81^{b}
20:1n-9	2.06 ± 0.40^{a}	2.41 ± 0.44^{c}	1.02 ± 0.20^{b}	1.08 ± 0.07^{b}
Sum MUFA	50.87 ± 10.88^{a}	48.89 ± 9.38^{a}	27.62 ± 4.45^{b}	25.88 ± 3.81^{b}
18:2n-6c	2.03 ± 0.35^{a}	2.09 ± 0.34^{a}	1.47 ± 0.20^{b}	1.50 ± 0.19^{b}
18:3n-3	nd	nd	26.94 ± 2.01	8.79 ± 1.98
20:4n-6	5.34 ± 1.02^{a}	5.31 ± 1.02^{a}	3.50 ± 0.81^{b}	3.52 ± 0.79^{b}
**20:3n-3**	**2.89 ± 1.07^{a}**	**2.10 ± 0.54^{a}**	**7.31 ± 1.69^{b}**	**11.58 ± 0.68^{c}**
20:4n-3	nd	nd	1.18 ± 0.33	nd
**20:5n-3**	**0.53 ± 0.17^{a}**	**0.71 ± 0.12^{a}**	**2.02 ± 0.54^{b}**	**0.43 ± 0.14^{ac}**
22:5n-3	0.83 ± 0.03^{a}	1.08 ± 0.03^{ab}	1.35 ± 0.33^{b}	0.62 ± 0.17^{c}
22:6n-3	4.07 ± 0.84^{a}	3.92 ± 0.73^{a}	2.77 ± 0.54^{b}	2.72 ± 0.55^{b}
Sum PUFA	15.69 ± 1.89^{a}	15.21 ± 1.43^{a}	46.53 ± 5.07^{b}	49.17 ± 0.48^{b}
EPA/DHA	0.13 ± 0.08	0.18 ± 0.07	0.72 ± 0.38	0.16 ± 0.06
ALA/LA	nv	nv	18.33 ± 2.11	19.24 ± 3.95

n=3; mean ± standard deviation. Values with different superscript letters within one row significantly differ with p-values < 0.05 with post-hoc multiple comparison test of Tukey. SFA: saturated fatty acids; MUFA: monounsaturated fatty acids; PUFA: polyunsaturated fatty acids; nd: not detectable (values below detection limits); nv: no value (due to values below detection limits).

Table S2 | Fatty acid composition (in % of total fatty acid methyl esters) of HepG2 cells treated for 48h with 40 µM of resveratrol (RSV) and additional 50 µM of BSA-bound α-linolenic acid (ALA) or 13 mg/ml of control BSA.

**Fatty acid**	**Control**	**RSV 40µM**	**Control + ALA**	**RSV 40µM + ALA**
14:0	1.81 ± 0.37	0.98 ± 0.61	1.73 ± 1.02	1.73 ± 0.30
16:0	19.69 ± 1.64	17.92 ± 2.32	17.58 ± 2.76	16.23 ± 1.15
18:0	9.78 ± 0.07^{a}	12.16 ± 0.76^{b}	7.53 ± 0.30^{c}	7.11 ± 0.40^{c}
20:0	0.53 ± 0.06	0.64 ± 0.08	0.43 ± 0.13	0.35 ± 0.10
Sum SFA	31.81 ± 10.93^{a}	31.70 ± 9.26^{a}	27.27 ± 4.42^{b}	25.42 ± 2.93^{b}
16:1n-7	5.53 ± 1.12	3.71 ± 0.99	4.53 ± 1.31	4.21 ± 1.22
18:1n-9c	23.97 ± 5.02^{a}	26.46 ± 5.65^{a}	11.37 ± 2.01^{b}	10.39 ± 1.78^{b}
18:1n-7c	22.86 ± 11.05^{a}	20.10 ± 10.19^{a}	12.36 ± 7.53^{b}	12.14 ± 6.92^{b}
20:1n-9	1.88 ± 0.29^{a}	2.43 ± 0.69^{a}	0.88 ± 0.13^{b}	0.87 ± 0.22^{b}
Sum MUFA	54.24 ± 11.38^{a}	52.69 ± 8.78^{a}	29.14 ± 6.25^{b}	27.61 ± 5.51^{b}
18:2n-6c	1.81 ± 0.58^{a}	1.92 ± 0.59^{a}	1.34 ± 0.26^{b}	1.15 ± 0.18^{b}
18:3n-3	nd	nd	27.15 ± 5.20	29.81 ± 4.39
20:4n-6	4.59 ± 1.66^{a}	4.95 ± 1.75^{a}	2.44 ± 0.41^{b}	2.62 ± 0.61^{b}
**20:3n-3**	**2.15 ± 1.09^{a}**	**2.54 ± 1.10^{a}**	**7.65 ± 0.89^{b}**	**10.58 ± 1.78^{c}**
20:4n-3	nd	nd	0.96 ± 0.17	nd
**20:5n-3**	**0.28 ± 0.08^{a}**	**0.49 ± 0.11^{a}**	**1.10 ± 0.38^{b}**	**0.16 ± 0.04^{c}**
22:5n-3	0.49 ± 0.12^{ab}	0 86 ± 0.40^{ab}	0.78 ± 0.21^{a}	0.37 ± 0.11^{b}
22:6n-3	3.76 ± 1.35^{a}	3.80 ± 1.41^{a}	1.66 ± 0.07^{b}	1.66 ± 0.22^{b}
Sum PUFA	13.08 ± 3.07^{a}	14.56 ± 5.27^{a}	43.07 ± 4.68^{b}	46.34 ± 6.32^{c}
EPA/DHA	0.07 ± 0.04	0.13 ± 0.07	0.66 ± 0.30	0.10 ± 0.05
ALA/LA	nv	nv	20.29 ± 2.06^{a}	25.84 ± 0.35^{b}

n=3; mean ± standard deviation. Values with different superscript letters within one row significantly differ with p-values < 0.05 with post-hoc multiple comparison test of Tukey. SFA: saturated fatty acids; MUFA: monounsaturated fatty acids; PUFA: polyunsaturated fatty acids; nd: not detectable (values below detection limits); nv: no value (due to values below detection limits).
